# Genome-Wide Association Study for Plant Height and Grain Yield in Rice under Contrasting Moisture Regimes

**DOI:** 10.3389/fpls.2016.01801

**Published:** 2016-11-29

**Authors:** Xiaosong Ma, Fangjun Feng, Haibin Wei, Hanwei Mei, Kai Xu, Shoujun Chen, Tianfei Li, Xiaohua Liang, Hongyan Liu, Lijun Luo

**Affiliations:** ^1^College of Plant Sciences & Technology, Huazhong Agricultural UniversityWuhan, China; ^2^Shanghai Agrobiological Gene CenterShanghai, China

**Keywords:** GWAS, plant height, grain yield, drought resistance, candidate genes

## Abstract

Drought is one of the vitally critical environmental stresses affecting both growth and yield potential in rice. Drought resistance is a complicated quantitative trait that is regulated by numerous small effect loci and hundreds of genes controlling various morphological and physiological responses to drought. For this study, 270 rice landraces and cultivars were analyzed for their drought resistance. This was done via determination of changes in plant height and grain yield under contrasting water regimes, followed by detailed identification of the underlying genetic architecture via genome-wide association study (GWAS). We controlled population structure by setting top two eigenvectors and combining kinship matrix for GWAS in this study. Eighteen, five, and six associated loci were identified for plant height, grain yield per plant, and drought resistant coefficient, respectively. Nine known functional genes were identified, including five for plant height (*OsGA2ox3, OsGH3-2, sd-1, OsGNA1*, and *OsSAP11/OsDOG*), two for grain yield per plant (*OsCYP51G3* and *OsRRMh*) and two for drought resistant coefficient (*OsPYL2* and *OsGA2ox9*), implying very reliable results. A previous study reported *OsGNA1* to regulate root development, but this study reports additional controlling of both plant height and root length. Moreover, *OsRLK5* is a new drought resistant candidate gene discovered in this study. *OsRLK5* mutants showed faster water loss rates in detached leaves. This gene plays an important role in the positive regulation of yield-related traits under drought conditions. We furthermore discovered several new loci contributing to the three investigated traits (plant height, grain yield, and drought resistance). These associated loci and candidate genes significantly improve our knowledge of the genetic control of these traits in rice. In addition, many drought resistant cultivars screened in this study can be used as parental genotypes to improve drought resistance of rice by molecular breeding.

## Introduction

Rice is one of the most important staple foods and plays an important role to ensure food safety. However, rice production consumes copious amounts of fresh water. In China, 49% of all fresh water resources are used for rice production (Zhang, 2007). Due to environmental damage and anomalous climatic variation, drought has become one of the main limiting factors for global grain production (Pennisi, 2008). Throughout Asia, 20% of all rice production areas are affected by drought per year (Gowda et al., 2011). This strengthens the disparity between supply and demand of rice production. Therefore, enhancing the drought resistance of rice is vital to reduce the effect of drought on rice production.

Drought, which is a soil water deficit, can result in insufficient moisture for a plant to adequately grow and complete its life cycle. Water shortage will seriously affect rice growth and lead to a series of physiological and biochemical changes. Many studies focused on plant drought resistance. These studies reported that drought usually leads to a delayed flowering time, restrained plant growth, and ultimately loss of yield in rice (Liu et al., 2005; Yue et al., 2005; Zou et al., 2007). The reduction in yield is due to a reduced spikelet number or reduced fertility of spikelet caused by drought stress (Ekanayake et al., 1989; Liu et al., 2010). In addition, drought can cause the change of antioxidant system and osmotic regulatory system in rice, accumulating antioxidants, and osmotic regulation substances (Ouyang et al., 2010).

With the development of molecular genetic technologies, more and more studies focused on the discovery of drought resistant genes and their function. These results are useful for breeders to develop drought resistant cultivars. QTL (quantitative trait loci) mapping used to be the main method to discover drought resistance loci. For example, many drought resistance-related QTLs were identified by using a recombinant inbred line (RIL) population derived from a cross of lowland *indica* rice Zhenshan 97 and upland *japonica* rice IRAT109 in previous studies (Yue et al., 2006; Liu et al., 2010). Based on the above drought resistance-related QTLs, 17 near-isogenic lines (NILs) were constructed and phenotypic variations of these NILs were investigated under drought and normal conditions, among them, *qFSR4* was fine mapped for spikelet number, flag leaf width and root volume, further analysis showed that *NARROW LEAF 1 (NAL1)* regulating leaf width was located in the *qFSR4* interval (Qi et al., 2008; Ding et al., 2011). Some studies have shown that *NARROW LEAF 1* can affect root development and drought resistance in rice (Fujita et al., 2013; Cho et al., 2014). In addition, root traits are very important traits for drought resistance in plant. *Dro1*, a major QTL involved in deep rooting of rice was cloned by fine-mapping, it can increase rice yield under drought conditions (Uga et al., 2011, 2013). And *qRT9*, a QTL controlling root thickness and root length was mapped an 11.5 kb interval in upland rice (Li et al., 2015). Although linkage analysis for drought resistance has made some achievements in rice, many QTLs/genes of drought resistance remain hidden. Due to a rapid development of sequencing techniques, genome-wide association studies (GWAS) became a new method for gene mining of target traits. Compared to QTL analysis, GWAS is based on natural populations, and can detect multiple alleles at the same site (Flint-Garcia et al., 2003). GWAS have widely been used in human genetic studies as well as in plants, such as *Arabidopsis*, maize, sorghum, and rice (Atwell et al., 2010; Huang et al., 2011; Tian et al., 2011; Li et al., 2012; Riedelsheimer et al., 2012; Morris et al., 2013; Chen et al., 2014; Wen et al., 2014; Yang et al., 2014, 2015; Wang et al., 2015; Yano et al., 2016). Identification of the allelic variation underpinning the phenotypic diversity in rice will result in enormous practical implications for rice breeding.

Genome-wide association study (GWAS) has been widely used for the genetic analysis of rice. Huang et al. (2010) constructed the first high-density haplotype map in rice via re-sequencing of 517 landraces and cultivars, and they identified 37 strong association loci for 14 agronomic traits via GWAS. Their study was considered as the coming of age for GWAS in rice (Clark, 2010). The genetic architecture of rice metabolism was dissected via GWAS, and five candidate genes were identified or annotated (Chen et al., 2014). Recently, a high-throughput phenotyping platform was developed for GWAS, achieving good results and further promoting GWAS for the dissection of genetic mechanisms for other traits (Yang et al., 2014).

In previous study, GWAS has been used to dissect drought resistance in maize (Lu et al., 2010; Wang et al., 2016; Zhang et al., 2016). However, a systematic examination of drought resistance in rice via GWAS was still called for. In this study, we conduct GWAS on a natural rice population under well watered and drought stress conditions, exploring the drought resistance of rice. The collection contains a wide range of genetic variation (Wu et al., 2015), highlighting its suitability for mapping studies aimed at detecting genetic variation and segregation in a diverse set of rice varieties. Using this strategy, 18, 5, and 6 loci could be associated with plant height, grain yield per plant, and drought resistant coefficient. Nine known genes were identified for three traits. The function of these genes is either directly or indirectly related to the three target traits. We also discovered new loci that contribute to these three traits and that were missed by previous studies. Among these, *OsRLK5* was preliminarily confirmed to positively regulate yield-related traits under drought condition.

## Materials and methods

### Plant material and field experiment

The plant material consisted of 270 accessions of rice landraces and cultivars, collected from Asia, Africa, and America. This population has previously been used in GWAS for mesocotyl elongation and ratio of deep rooting (Lou et al., 2015; Wu et al., 2015). All accessions were tested under two water regimes: well watered and drought stress, in a drought resistance screening facility (Luo, 2010) at the Baihe Experimental Station of the Shanghai Agrobiological Gene Center (31°15′N, 121°10′E, 4 m altitude) in 2011 and 2012. The plant material was arranged by single factor randomized block design. Seeds derived from a single plant, from which the genomic DNA was extracted for sequencing, was used for field trials. Staged sowing was used according to growth durations. There were 22 hills per row with a space of 18 cm between rows and 16 cm between hills. Fertilizer application and pest control were identical to normal field management. The method for drought treatment was the same as reported in our previous study (Liu et al., 2005). Drought stress was implemented at the early booting stage and lasted for a total of 35 days. During the treatment, drip irrigation was provided to keep the plants growing well in the well-water treatment regime every day and stop watering in drought stress regime.

### Measurement of soil water content, plant traits, and statistical analysis

Soil moisture content was measured every 3 days for both drought stress regime and irrigational regime during drought stress. Plant height (PH) was investigated before harvest and grain yield per plant (GY) was investigated after harvest. The drought resistant coefficient (DRC) was calculated as the ratio of the grain yield per plant under drought stress regime to the grain yield per plant under water regime. Statistic analysis was conducted using SPSS software (version 19.0). Mixed model was used for ANOVA of phenotypic data. Genotype and treatment were treated as fixed factor while year was treated as a random factor. Type Π sums of squares were used for ANOVA for unbalanced data in **Table 2** (Langsrud, 2003).

### Genotype

Genomic DNA (gDNA) was extracted from a single plant and used for sequencing. A total of 270 accessions were genotyped via re-sequencing and using an Illumina HiSeq2000. Among these, genotypic data of 101 accessions were generated by Shanghai Agrobiological Gene Center, and the genotypic data of the remaining 169 accessions were provided by the Huazhong Agricultural University (Chen et al., 2014; Yang et al., 2014). Paired-end sequence reads were mapped to a rice reference genome sequence of *japonica* cv. *Nipponbare* (MSU v6.1) using the software BWA, then used for SNP identification, following the procedures described by Wu et al. (2015).

### GWAS analysis

The genome-wide association mapping was conducted via the efficient mixed-model association (EMMA) method using the GAPIT software package in R (Lipka et al., 2012). For this study, a total of 1019,883 SNP markers were used for GWAS. 144,995 SNPs, with less than 10% missing data in this natural population, were used for kinship calculation among individuals and principal component analysis (PCA) to adjust the population structure. The genome-wide threshold was set at *p* = 9.81E-07, calculated via the formula: 1/total number of SNPs, which was widely used in plant GWAS studies (Wen et al., 2014; Wang et al., 2015; Bai et al., 2016). We furthermore evaluated the extent of local LD (linkage disequilibrium) for each significant SNP. The extended region, where LD between nearly SNPs and lead SNP (with the lowest *p*-value) decayed to *r*^2^ = 0.6, was defined as the local LD-based QTL interval (Yano et al., 2016). To test whether the significant associated loci respond to drought stress, we performed a two-way ANOVA for each significant locus to test the significance of the interactive effect between locus and water status (normal water and drought stress). The set of loci with significant interactions with the water status were defined as QTL responsive to drought stress (Zhang et al., 2016).

### The deletion mutants

The CRISPR/Cas9 method was used to generate deletion mutants for the verification of candidate gene function. The mutant of *OsRLK5* was obtained via the CRISPR/Cas9 method as described by Zhang et al. (2014), using the target sequence GAAAGATCCCGAAGTGGATA**TGG**. We obtained homozygous individuals with 1 bp deletion within target sequences (GAAAGATCCCGAAGT-GATA**TGG**) at the CDS region.

The mutant of *OsGNA1* was gained via the CRISPR/Cas9 method as described by Ma et al. (2015), with the target sequence of GGGGCACGTCGAGGACGTCG**TGG**. Several individual homozygous or heterozygous plants were obtained. The homozygous mutants had the target sequence GGGGCACGTCGAGGAC-TCG**TGG**, and those homozygous and heterozygous mutants with 1 bp deletion at the CDS region were used in further experiments. The primers used for identification of mutants and haplotype analysis of *OsGNA1* were listed in Table S1.

### Sequence analysis of *OsGNA1*

We sequenced the promoter and coding region of *OsGNA1* in the natural population. Sequence alignment was conducted with ClustalX 1.83. Cluster analysis was conducted via MEGA 6.0 (Tamura et al., 2013). LD analysis was conducted using Haploview 4.2 (http://www.broad.mit.edu/mpg/haploview/; Barrett et al., 2005).

## Results

### The change of soil water content during drought stress in 2011 and 2012

Drought treatment lasted for a total of 35 days. In this period, the absolute water content of the soil declined from 23.03 to 7.78%. Under the drip irrigation regime in 2011, values were always higher than 18.88%. The absolute water content of the soil declined from 22.98 to 8.07% under drought stress regime, while always remaining higher than 19.01% under the drip irrigation regime in 2012 (Figure S1). The change of soil water content showed a similar trend in both years.

### Phenotype statistics and analysis of variance of traits

The descriptive statistics for three traits are shown in Table 1. The coefficient of variation among different genotypes ranged from 25.70 to 28.98, 30.51 to 84.49, and 69.04 to 85.09% for PH, GY, and DRC, respectively. The ANOVA results revealed significant differences of all three traits for genotypes, treatments, and years (Table 2). These results indicate large phenotypic variation in this natural population, possibly increasing the chance to discover candidate genes for related traits via genetic analysis.

**Table 1:** **The descriptive statistics for PH, GY, and DRC in the natural population**.

**Year**	**Treatment**	**Trait**	**No**.	**Range**	**Mean ± SD**	**CV (%)**
2011	Normal water	PH (cm)	260	60.88 ~ 206.88	118.36±30.42	25.70
		GY (g)	234	2.6989 ~ 23.7819	9.7533±3.7693	38.65
	Drought stress	PH (cm)	260	55.33 ~ 176.25	98.00±27.34	27.90
		GY (g)	227	0.7457 ~ 19.7537	4.4528±2.800	62.88
		DRC	227	0.0877 ~ 2.0962	0.5245±0.3621	69.04
2012	Normal water	PH (cm)	239	70.22 ~ 219.89	123.12±31.85	25.87
		GY (g)	239	2.0739 ~ 18.2551	10.6080±3.2370	30.51
	Drought stress	PH (cm)	239	44.25 ~ 184.00	103.79±30.08	28.98
		GY (g)	235	0.0392 ~ 22.5882	5.6622±4.7842	84.49
		DRC	235	0.0036 ± 2.2102	0.5699±0.4849	85.09

CV, Coefficient of Variation.

**Table 2:** **ANOVA of traits based on data from experiments in 2011 and 2012**.

**Trait**	**Variation**		**SS**	**df**	**MS**	***F***	***P*-value**
GY	Genotype	Hypothesis	5099.370	245	20.814	2.161	3.236E-09
		Error	2178.786	226.265	9.629		
	Treatment	Hypothesis	6087.460	1	6087.460	3530.116	2.766E-138
		Error	382.825	222	1.724		
	Year	Hypothesis	256.459	1	256.459	26.441	5.890E-07
		Error	2187.709	225.556	9.699		
	Genotype × Treatment	Hypothesis	5274.878	239	22.071	12.799	2.256E-66
		Error	382.825	222	1.724		
	Genotype × Year	Hypothesis	2182.103	226	9.655	5.599	2.414E-34
		Error	382.825	222	1.724		
PH	Genotype	Hypothesis	810537.257	269	3013.150	26.387	2.236E-101
		Error	26011.567	227.791	114.191		
	Treatment	Hypothesis	98168.876	1	98168.876	1017.069	5.230E-86
		Error	22006.858	228	96.521		
	Year	Hypothesis	4051.575	1	4051.575	35.486	9.645E-09
		Error	26049.460	228.156	114.174		
	Genotype × Treatment	Hypothesis	32417.674	269	120.512	1.249	4.169E-02
		Error	22006.858	228	96.521		
	Genotype × Year	Hypothesis	26136.996	229	114.135	1.182	1.030E-01
		Error	22006.858	228	96.521		
DRC	Genotype	Hypothesis	80.349	239	0.336	17.245	1.047E-78
		Error	4.308	221	0.019		
	Year	Hypothesis	0.350	1	0.350	17.940	3.345E-05
		Error	4.308	221	0.019		

SS, sum of squares; df, degree of freedom; MS, mean square.

### Genome-wide association study

Principal component analysis (PCA) analysis revealed two divergent groups belonging to two subspecies of cultivated rice (Figure S2), the *indica* subspecies and the *japonica* subspecies, respectively. This is consistent with known information of the rice germplasm (Huang et al., 2010).

We controlled population structure by setting top two eigenvectors and combining kinship matrix for GWAS in this study. A total of 18, 5, and 6 loci were detected for plant height, grain yield per plant, and drought resistant coefficient, respectively (Table 3).

**Table 3:** **Summary of GWAS loci and their interactions with water status**.

**Loci**	**QTLs**	**Traits**	**Lead SNP**	**Chr**	***P*-value**	**MAF**	**Var%**	***P*-value (G × E)**
1	*qDRC2.1*	DRC_2011	sf0207287189	2	3.66E-07	0.15	17.36	
2	*qDRC2.2*	DRC_2011	sf0225263251	2	3.54E-07	0.19	10.70	
3	*qDRC7*	DRC_2011	sf0707946689	7	8.55E-07	0.11	9.38	
4	*qDRC8*	DRC_2011	sf0804114951	8	3.14E-09	0.06	21.12	
4	*qDRC8*	DRC_2012	sf0804114951	8	8.31E-08	0.06	14.99	
5	*qDRC10*	DRC_2012	sf1009214335	10	9.14E-07	0.30	23.56	
6	*qDRC11*	DRC_2012	sf1127965754	11	8.88E-07	0.09	18.18	
7	*qGY1*	GY_2011_W	sf0127752061	1	1.24E-07	0.42	45.48	*1.39E-17*
8	*qGY5*	GY_2011_W	sf0506803017	5	5.47E-07	0.43	45.61	*4.95E-17*
4	*qGY8*	GY_2011_D	sf0804114951	8	4.98E-11	0.06	28.03	*3.68E-07*
4	*qGY8*	GY_2012_D	sf0804114951	8	1.95E-08	0.06	16.95	*4.95E-08*
9	*qGY9*	GY_2011_D	sf0920270490	9	2.42E-08	0.07	25.03	*1.04E-03*
10	*qGY10*	GY_2011_D	sf1011166180	10	3.78E-10	0.07	27.03	*8.10E-04*
10	*qGY10*	GY_2012_D	sf1011166180	10	7.57E-08	0.07	15.64	*9.01E-05*
11	*qPH1.1*	PH_2012_D	sf0123707238	1	6.42E-07	0.14	31.58	0.749297
12	*qPH1.2*	PH_2011_W	sf0124424308	1	3.69E-07	0.06	7.11	0.867463
13	*qPH1.3*	PH_2011_D	sf0132497792	1	1.85E-07	0.14	13.02	0.440677
13	*qPH1.3*	PH_2011_W	sf0132455873	1	8.15E-08	0.12	8.85	0.969805
13	*qPH1.3*	PH_2012_D	sf0132455873	1	1.76E-07	0.12	11.12	0.710762
13	*qPH1.3*	PH_2012_W	sf0132455873	1	6.25E-07	0.12	11.97	0.710762
14	*qPH1.4*	PH_2011_D	sf0138428598	1	4.21E-08	0.40	18.96	0.395289
14	*qPH1.4*	PH_2011_W	sf0138427506	1	1.60E-07	0.44	11.17	0.53344
14	*qPH1.4*	PH_2012_D	sf0138426095	1	9.87E-09	0.44	16.13	0.659011
14	*qPH1.4*	PH_2012_W	sf0138427506	1	2.48E-07	0.44	11.73	0.664911
15	*qPH2*	PH_2011_D	sf0219187099	2	7.76E-08	0.37	24.35	0.408649
15	*qPH2*	PH_2012_D	sf0219200822	2	4.68E-08	0.37	24.70	0.768741
16	*qPH3*	PH_2012_D	sf0336180940	3	4.38E-07	0.07	8.28	0.528972
17	*qPH4*	PH_2012_D	sf0402075595	4	1.79E-07	0.09	23.64	0.496798
17	*qPH4*	PH_2012_W	sf0402075595	4	3.84E-08	0.09	26.19	0.496798
18	*qPH6*	PH_2012_D	sf0610068717	6	3.99E-07	0.21	11.48	0.88451
18	*qPH6*	PH_2012_W	sf0610068717	6	7.59E-07	0.21	11.04	0.88451
19	*qPH7.1*	PH_2012_D	sf0725741676	7	2.82E-08	0.26	22.71	0.83416
19	*qPH7.1*	PH_2012_W	sf0725741676	7	6.15E-08	0.26	22.54	0.83416
20	*qPH7.2*	PH_2011_W	sf0726948071	7	1.17E-07	0.36	18.95	0.672361
21	*qPH8.1*	PH_2012_D	sf0825091299	8	2.68E-08	0.31	13.53	0.683081
22	*qPH8.2*	PH_2012_W	sf0825517102	8	1.38E-07	0.12	12.95	0.740841
23	*qPH9.1*	PH_2011_W	sf0904228678	9	4.51E-09	0.11	13.23	0.349388
23	*qPH9.1*	PH_2012_D	sf0904275629	9	3.80E-07	0.12	14.40	0.521177
23	*qPH9.1*	PH_2012_W	sf0904228678	9	2.60E-09	0.10	16.50	0.369914
24	*qPH9.2*	PH_2012_D	sf0918830330	9	2.60E-08	0.32	12.77	0.672421
24	*qPH9.2*	PH_2012_W	sf0918839813	9	7.64E-07	0.19	21.81	0.74589
25	*qPH10*	PH_2011_W	sf1003062910	10	1.12E-08	0.14	7.05	0.540357
25	*qPH10*	PH_2012_D	sf1003193980	10	6.13E-08	0.13	10.13	0.804655
25	*qPH10*	PH_2012_W	sf1003193980	10	3.36E-08	0.13	10.31	0.804655
26	*qPH11*	PH_2012_W	sf1122822808	11	2.60E-07	0.13	27.79	0.309761
27	*qPH12.1*	PH_2012_W	sf1203637715	12	9.15E-08	0.27	16.60	0.307528
28	*qPH12.2*	PH_2012_D	sf1224306015	12	5.83E-07	0.11	3.12	0.715938

Chr, chromosome; MAF, minor allele frequency; Var%, phenotype variation explained by each locus; P-value (G × E), the significance of the interaction between the loci and water status that was estimated by a two-way ANOVA analysis, the italics value indicates the locus response to water status is significant (p < 0.05).

**Figure 1:**
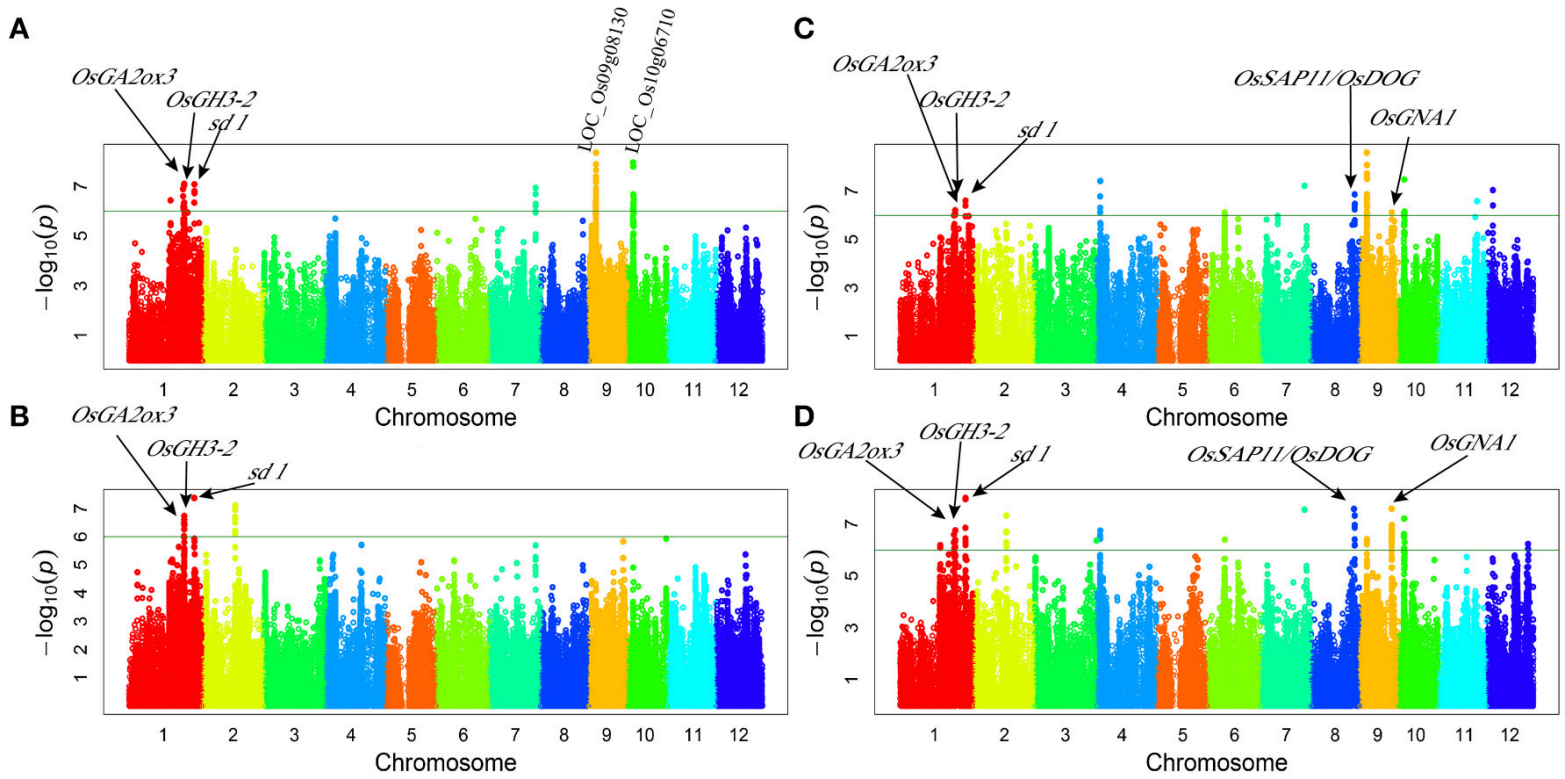
**Genome-wide manhattan plots of association mapping for plant height. (A)** plant height under normal water condition in 2011; **(B)** plant height under drought stress condition in 2011; **(C)** plant height under normal water condition in 2012; **(D)** plant height under drought stress condition in 2012.

Genome-wide association study (GWAS) for plant height: 13 associated loci were detected under normal water conditions and 13 associated loci under drought stress while eight loci were detected under both conditions (Figure 1, Table 3). These QTLs explained a range from 3.12 to 31.58% of phenotypic variation. The associated SNP sf0123707238 on chromosome 1 provided the largest contribution to PH variation. *qPH1.3* and *qPH1.4* were detected under both normal water and drought stress conditions in both years, explaining the range from 8.85 to 18.96% of the phenotypic variation. Additionally, two-way ANOVA revealed that all loci that were detected for plant height showed no significant interaction between locus and water status, indicating that these QTLs of plant height were not responsive to drought stress.

The peak signal of the GWAS loci often appeared in the region near but not within the known genes (Huang et al., 2010). We found the similar cases of five known genes involved in plant height: *OsGA2ox3* (Lo et al., 2008), *OsGH3-2* (Du et al., 2012), *sd-1* (Spielmeyer et al., 2002), *OsDOG/OsSAP11* (Giri et al., 2011; Liu et al., 2011), and *OsGNA1* (Jiang et al., 2005). These genes were located within or nearby four QTLs intervals and were identified for PH via GWAS (Figure 1, Table 4). Additionally, according to genome annotation information (MSU 6.1) and KEGG pathway database, LOC_Os09g08130 and LOC_Os10g06710 related to the indole alkaloid biosynthesis pathway, located near *qPH9.1* and *qPH10*, respectively (Table 3).

**Table 4:** **The GWAS hits associated to known genes**.

**Traits**	**GWAS locus (msu6.1)**	***P*-value**	**Known gene**	**Distance (kb)**
PH_2011_W	sf0131767145	2.50E-07	*OsGA2ox3* (LOC_Os01g55240)	27
PH_2011_W	sf0132127194	8.90E-07	*OsGH3-2* (LOC_Os01g55940)	93
PH_2012_D	sf0138416827	3.58E-07	*sd-1* (LOC_Os01g66100)	35
PH_2012_D	sf0825091299	2.68E-08	*OsDOG/OsSAP11* (LOC_Os08g39450)	140
PH_2012_D	sf0918825447	4.18E-07	*OsGNA1* (LOC_Os09g31310)	within
GY_2011_W	sf0506803017	5.47E-07	*OsCYP51G3* (LOC_Os05g12040)	81
GY_2011_D	sf0920173743	5.85E-07	*OsRRMh* (LOC_Os09g34070)	60
DRC_2011	sf0207150775	9.62E-07	*OsPYL2* (LOC_Os02g13330)	39
DRC_2011	sf0225263251	3.54E-07	*OsGA2ox9* (LOC_Os02g41954)	65

**Figure 2:**
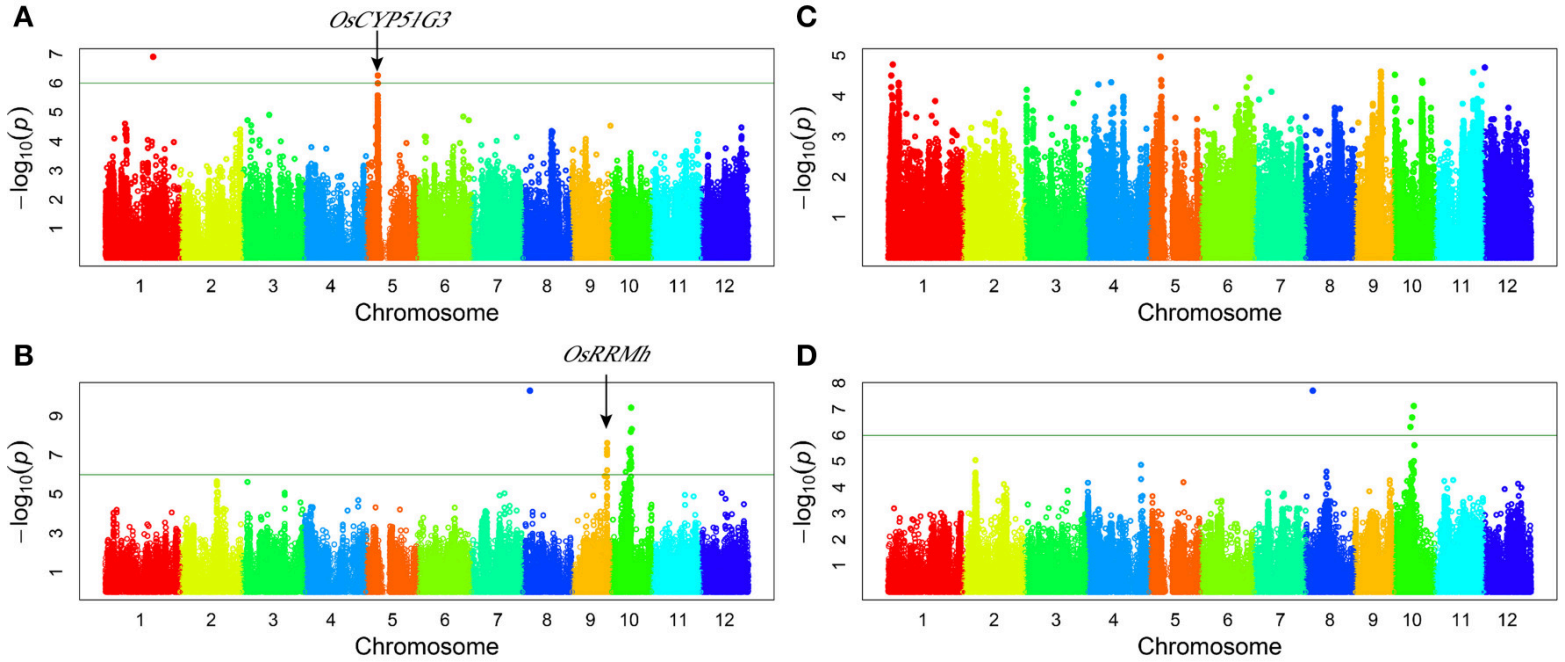
**Genome-wide manhattan plots of association mapping for grain yield per plant. (A)** grain yield under normal water condition in 2011; **(B)** grain yield under drought stress condition in 2011; **(C)** grain yield under normal water condition in 2012; **(D)** grain yield under drought stress condition in 2012.

Genome-wide association study (GWAS) for grain yield: a total of five QTLs were detected for grain yield per plant. Three QTLs (*qGY8, qGY9*, and *qGY10*) were detected under drought stress and two QTLs (*qGY1* and *qGY5*) were detected under normal water conditions (Figure 2, Table 3). These QTLs explained the range from 15.64 to 45.61% of phenotypic variation. The associated SNP sf0506803017 on chromosome 5 contributed the highest to GY variation (Table 3). *qGY8* and *qGY10* were detected for GY under drought stress in both 2011 and 2012. In addition, the two-way ANOVA revealed all loci detected for grain yield showing a significant interaction between locus and water status, indicating that these QTLs of grain yield were likely responsive to drought stress.

*OsCYP51G3* (LOC_Os05g12040) was found to be located near the lead SNP sf0506803017 (Figure 2A, Table 4) and involved in steroid biosynthesis, regulating plant height and seed setting rate (Xia et al., 2015). *OsRRMh* (LOC_Os09g34070) was 157 kb away from the significant association signal (sf0920270490) (Figure 2B, Table 4) and has been reported to regulate fertility rate and number of spikelet per panicle (Liu and Cai, 2013).

**Figure 3:**
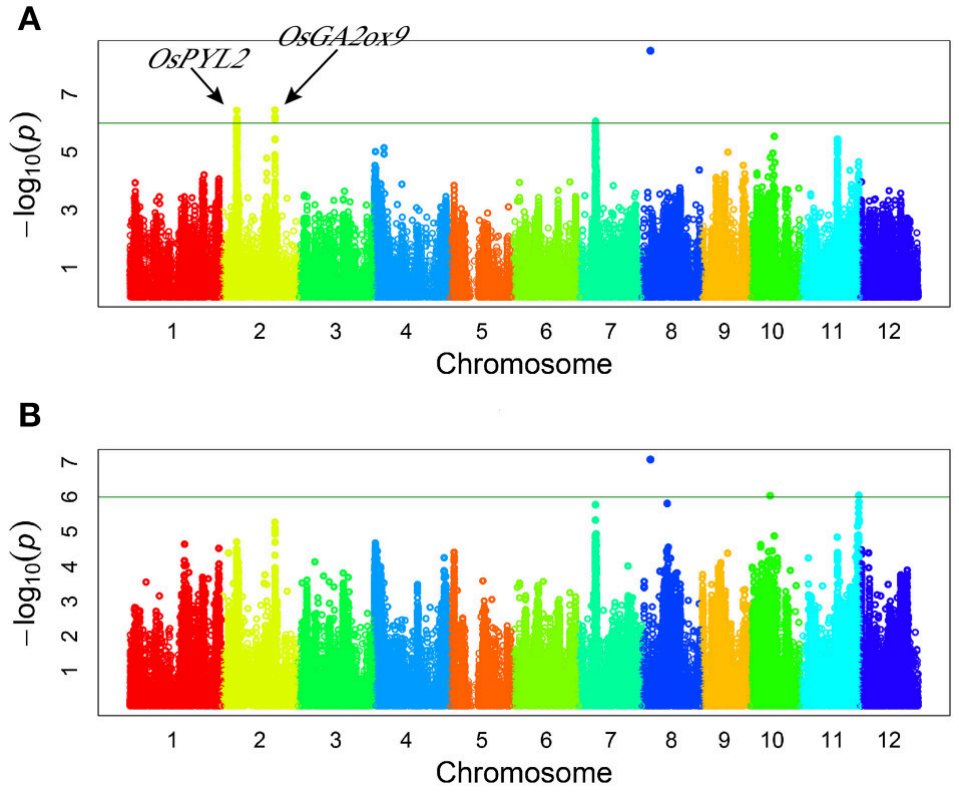
**Genome-wide manhattan plots of association mapping for drought resistant coefficient. (A)** DRC in 2011; **(B)** DRC in 2012.

Genome-wide association study (GWAS) for drought resistant coefficient: six QTLs were detected for DRC, explaining a range of the phenotypic variation from 9.38 to 23.56% (Figure 3, Table 3). Since the DRC reflected relative yield between drought stress and normal water condition, these six QTLs for DRC should be involved in the response to drought stress. Two known genes are close to the association intervals (Figure 3), i.e., *OsPYL2* (Tian et al., 2015) near the lead SNP sf0207287189 and *OsGA2ox9* (Lo et al., 2008) near the lead SNP sf0225263251.

### Candidate genes involved in drought-response loci

The purpose of rice drought resistance breeding is to improve grain yield under drought conditions. DRC reflects the yield changes (mostly decreases) of plants suffering from drought stress. A total of 162 candidate genes were discovered based on 10 GWAS loci (QTL interval, *r*^2^ of LD > 0.6), involved in response to drought stress as well as RNA-seq data under contrasting water status (unpublished data) (Table S2). Among them, 63 genes were up-regulated more than 2-fold and 99 genes were down regulated to less than half their expression. The expression levels of most candidate genes under drought stress conditions tended to return to normal levels after re-watering.

**Figure 4:**
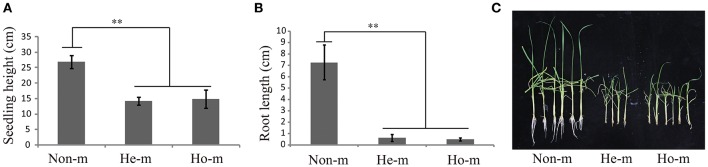
**Analysis of seeding height and root length for the mutant of *OsGNA1*. (A)** seedling height in T0 mutant plants. **(B)** root length in T0 mutant plants. Values represent mean ± SD. Non-m, non-mutant (*n* = 13), He-m, heterozygous mutants (*n* = 4), Ho-m, homozygous mutants (*n* = 6). “^**^” represent significances at *p* < 0.01.

*OsGNA1* plays an important role for plant height and root length. It is noticeable that the sf0918825447 at the promoter of *OsGNA1* (LOC_Os09g31310) was detected for plant height under drought stress conditions with a *p*-value of 4.18E-07 in 2012. It was 5 kb away from the lead SNP (sf0918830330) for plant height under drought stress. It remains unclear whether *OsGNA1* regulates plant height, although it has been shown to regulate root development (Jiang et al., 2005). To confirm whether *OsGNA1* regulates plant height, we generated a gRNA construct and introduced it into the *Nipponbare* to knock out the *OsGNA1* gene via a CRISPR/Cas 9 strategy (Ma et al., 2015). Several homozygous or heterozygous mutant plants were obtained. The seedling height and root length of both heterozygous and homozygous mutants were significant shorter compared to non-mutant transgenic plants (Figure 4). These results demonstrate that *OsGNA1* positively regulates plant height and root length. Elevated expression levels were beneficial to plant height and root length.

**Figure 5:**
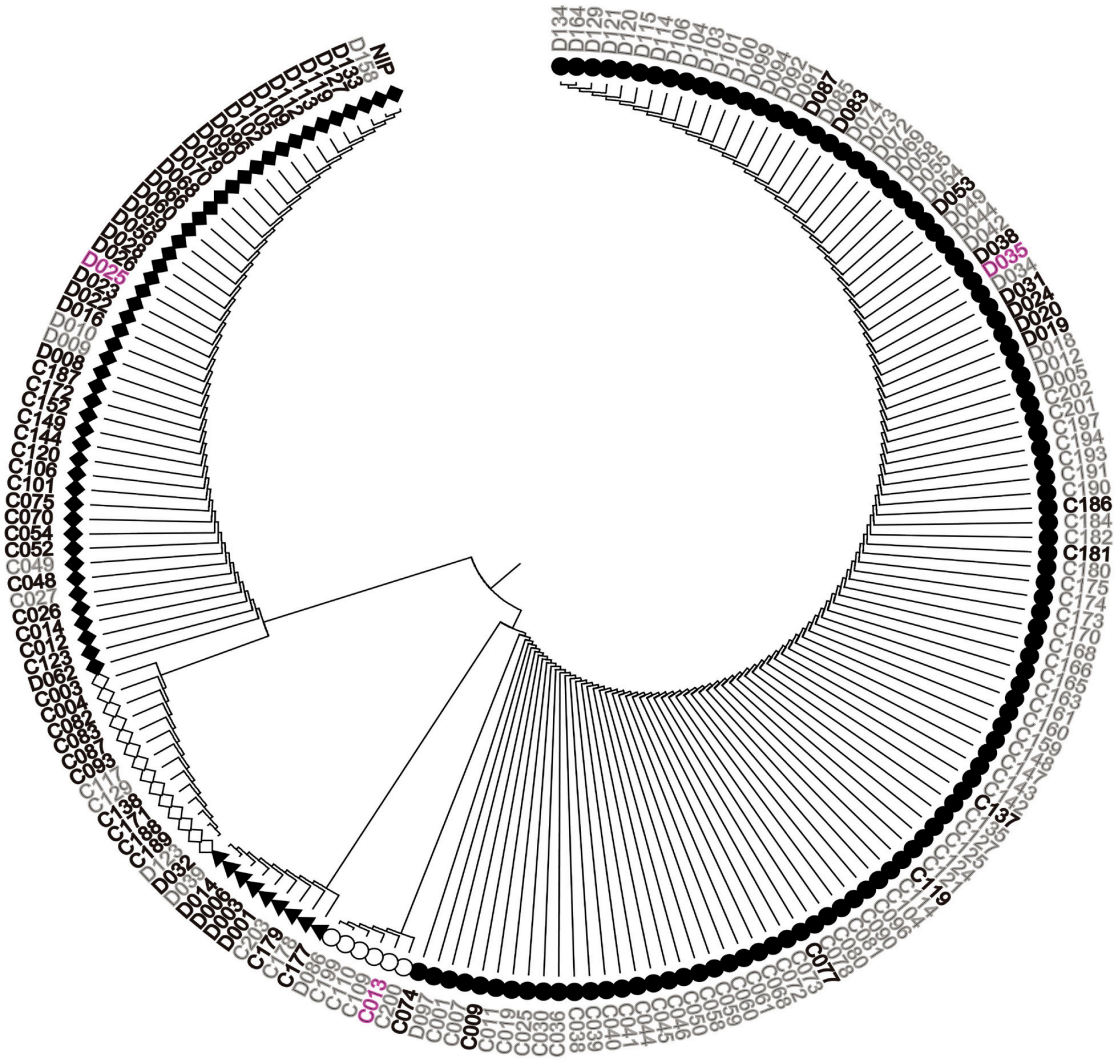
**Cluster analysis of *OsGNA1***. Black font represents japonica, gray font represents indica, purple font represents aus. NIP = Nipponbare.

**Figure 6:**
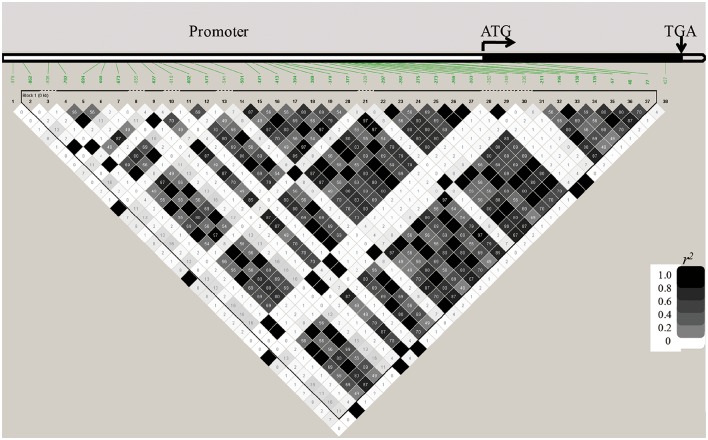
**LD analysis of *OsGNA1***.

We sequenced *OsGNA1* from −891 to 748 bp in 184 accessions of the natural population and identified 38 polymorphic loci (36 SNPs and 2 indels) (Table S3). Among them, one indel (+48) and two SNPs (+77, +427) at the CDS region caused insertion/deletion of two amino acids, substitution of Pro (CCG) to Leu (CTG), and Pro (CCG) to Ser (TCG), respectively. Cluster analysis divided 184 accessions into five groups (Figure 5). LD analysis of 38 polymorphism loci is depicted in Figure 6. We performed haplotype analyses based on 38 polymorphism loci of *OsGNA1* and classified the genotype into five haplotypes (Hap) (Table S3). Hap1 and Hap5 mainly contained the *indica* subpopulation, while Hap2 mainly contained the *japonica* subpopulation. At adult stage under drought stress Hap2 had the lowest PH, and Hap5 had the highest PH and highest DRC. At seedling stage, Hap5 had the longest root length (Table S3). The one amino acid mutation caused by +427 SNP only existed in Hap5 (Table S3).

**Figure 7:**
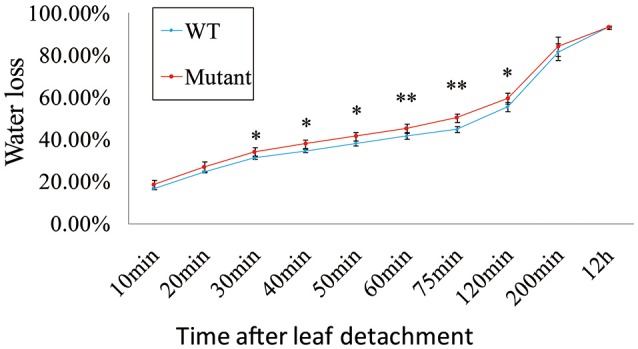
**Water loss in the mutant of *OsRLK5***. Values represent mean ± SD, *n* = 5. “^*^” and “^**^” represent significances at *p* < 0.05, *p* < 0.01, respectively.

**Figure 8:**
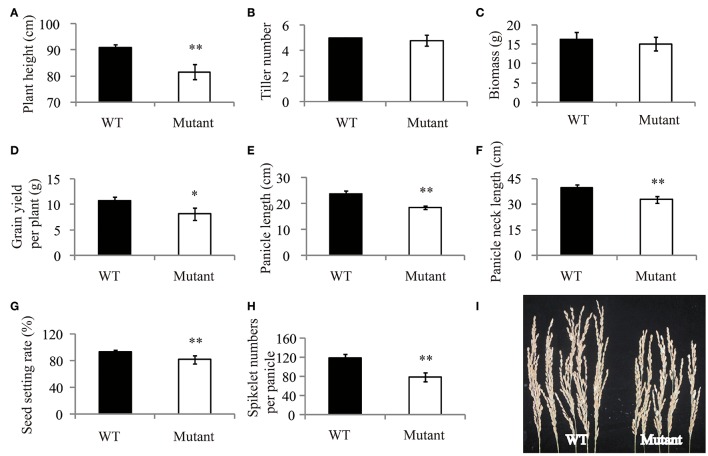
**Drought resistance assay of *OsRLK5* under drought stress condition**. Plant height **(A)**, tiller number **(B)**, biomass **(C)**, grain yield per plant **(D)**, panicle length **(E)**, panicle neck length **(F)**, seed setting rate **(G)**, and spikelet numbers per panicle **(H)** were measured under drought stress condition. **(I)**, the panicle of wild-type Nipponbare (WT) and transgenic plant (Mutant). Values represent mean ± SD, *n* = 5. “^*^” and “^**^” represent significances at *p* < 0.05, *p* < 0.01, respectively.

Mutation of *OsRLK5* reduced the tolerance of plants to drought stress. *OsRLK5* (LOC_Os02g13430) was a differentially expressed gene in the *qDRC2.1* interval (Table S2), we generated a gRNA construct that was introduced into the *Nipponbare* to knock out *OsRLK5* via CRISPR/Cas 9 technology (Zhang et al., 2014). Rate of water-loss of detached leaves and grain yield related traits were identified in both homozygous mutants and wild-type plants. As depicted in Figure 7, the mutant plants lost water faster than the wild-type plants. Compared to wild-type plants, plant height, panicle length, panicle neck length, seed-setting rate, spikelet numbers per panicle, and grain yield per plant were significantly reduced in the mutant plants under drought stress conditions (Figure 8). These results demonstrate that *OsRLK5* is responsible for the GY under drought stress.

Additionally, using genome-wide association study for eigenvectors (EigenGWAS; Chen et al., 2016), five SNPs in the loci of *OsRLK5* were significantly detected for the third eigenvector, implying this gene was under selection within this population. None or few SNPs were detected for all three largest eigenvectors for *OsGNA1* and eigenvector 1 and 2 for *OsRLK5* (Table S4). Roughly, eigenvector 1 is responding to the population stratification of *indica* and *japonica* subspecies. Further analysis is needed to understand the selective effect on *OsRLK5* gene among subgroups determined by eigenvector 3.

## Discussion

Drought is one of the main abiotic stresses affecting the production of most field crops. Drought stress can occur at any crop growth stage and can affect productivity to variable degrees depending on the onset time, duration, and intensity of drought. Drought stress typically suppresses plant growth at the vegetable stage and has been reported to directly reduce production at the reproductive stage (Yue et al., 2005, 2006). Agronomists and breeders have made significant progress in improving the drought resistance of crops, however, the genetic and molecular basis for drought resistance in crops remains largely unclear. In this study, drought resistance of the rice germplasm was evaluated using contrasting soil moisture regimes. Combining high density SNPs and phenotypes of this germplasm, we conducted GWAS for plant height, grain yield per plant, and drought resistant coefficient. Five, two, and two known genes for the same three traits were found within or close to the associated loci, respectively. These associated loci and candidate genes could enhance the knowledge for understanding the mechanisms underlying rice drought resistance and could be used in improvement of drought resistance of rice via molecular breeding. In addition, many drought resistant germplasm accessions were obtained in this study that should be valuable in rice breeding program facing water scarcity.

Compared to linkage analysis, GWAS greatly accelerated both speed and accuracy of locating QTL or candidate genes. For example, *OsSPL13* has been quickly identified based on genome-wide association study for the grain size and expression profiling in panicles between small-grain and large-grain varieties. Further analysis indicated that *OsSPL13* positively regulates cell size in the grain hull, resulting in enhanced grain length and yield in rice (Si et al., 2016). In this study, nine reported functional genes were found to be near the significant GWAS loci (Table 4). The function of these genes is consistent with their associated traits. The discovery of the novel drought resistant gene *OsRLK5* and the new functions of *OsGAN1* in this study further support GWAS as a viable approach to quickly identify new genes that are contributing to the complex traits.

Numerous factors affect plant height of rice. Gibberellin (GA) is one of the most important determinants (Monna et al., 2002; Sasaki et al., 2002; Spielmeyer et al., 2002). In this study, several candidate genes (*SD-1, OsGA2ox3*, and *OsDOG*), controlling rice plant height were involved in synthesis or signal transduction of GA. For example, *SD-1* encodes a gibberellin 20-oxidase, a key enzyme in the biosynthesis of gibberellin catalyzing (GA53 → GA44 → GA19 → GA20) (Monna et al., 2002; Sasaki et al., 2002; Spielmeyer et al., 2002). Interestingly, the GWAS approach also found that *SD-1* is important for plant height under drought stress condition (Figure 1), suggesting that *SD-1* may be involved in drought resistance. *OsGA2ox3* was also involved in the GA metabolic pathway, and it has been reported that *OsGA2ox3* regulates plant height and tiller number (Lo et al., 2008). Another candidate gene for rice height, *OsDOG*, has been reported to act as a new dynamic equilibrium regulator for the gibberellin metabolism. It has furthermore been reported that *OsDOG* negatively regulates cell elongation and plant height in rice (Liu et al., 2011). *OsSAP11* was an A20/AN1 zinc-finger gene and contained stress-associated proteins. Over-expression of *OsSAP11* can enhance the tolerance to water deficit and salt stress in transgenic *Arabidopsis* plants (Giri et al., 2011). *OsDOG*/*OsSAP11* was identified within the important locus for plant height, especially as a significant signal with lower *p*-value under drought stress conditions.

Auxin plays an important role in plant height. In this study, *OsGH3-2* was identified at the *qPH1.2* interval, encoding an enzyme catalyzing the IAA conjunction to amino acids. *OsGH3-2* has been reported to be involved in the regulation of plant height, root and stomatal development, as well as in the modulation of ABA level and drought resistance (Du et al., 2012). Two more candidate genes (LOC_Os09g08130 and LOC_Os10g06710) that were detected for plant height are also related to the auxin metabolism. LOC_Os09g08130 was annotated as indole-3-glycerol phosphate synthase and was located upstream of the indole alkaloid biosynthesis pathway. LOC_Os10g06710 was annotated as amidase, involved in the metabolic pathway of indole acetic acid. Indole acetic acid is an important phytohormone and plays a crucial role in cell division, differentiation, and elongation, in root development and plant height regulation (Petersson et al., 2009; Du et al., 2012; Lu et al., 2015).

In particular, a significant SNP (sf0918825447) with *p*-value of 4.18E-07 for plant height is located at the promoter region of *OsGNA1*. The associated peak signal was detected with a lower *p*-value under drought stress condition compared to normal water condition, implying its contribution to drought resistance. A previous study demonstrated *OsGNA1* to play an important role in root development. Compared to wild type rice plants, mutant *gna1* plants exhibited reduced root elongation, shorter taproot, lateral root and root hairs (Jiang et al., 2005). Similarly, the mutant of *OsGNA1* exhibited shorter root length and seedling height compared to wild type plants in this study. These results suggest that *OsGNA1* can regulate plant height and drought resistance. A haplotype analysis revealed that Hap5 was likely to improve drought resistance in rice because Hap5 had the highest PH at the adult stage under drought stress and had the longest maximum root length at seedling stage. Moreover, Hap5 had the highest DRC (Table S3).

In this study, the candidate genes for drought responsive associated loci were discovered together with a set of RNA-Seq data for rice plants under contrasting moisture regimes (unpublished). A total of 162 genes showed significantly different expression levels in leaves between drought treated and normal water treated plants. Two functional genes, *OsPYL2* and *OsGA2ox9* were discovered in this study. *OsGA2ox9* was involved in GA metabolic pathways, and a previous study reported it to control plant height, tiller number, and root length in rice (Lo et al., 2008). Roots play an important role for absorbing water and nutrients. Plants can benefit from longer roots for water uptake from deeper soil layers as drought stress occurs. These above results imply that *OsGA2ox9* was likely affecting drought resistance via regulation of root growth in rice. *OsPYL2* was reported to be an ABA receptor (Tian et al., 2015). ABA is involved in the adaptation of plants to abiotic stress. The combination of ABA and the ABA receptor can activate the ABA signal transduction and trigger the corresponding physiological reaction, including stomatal closure (Cutler et al., 2010; Kim et al., 2010). Thus, *OsPYL2* is likely to regulate stomatal behavior to reduce water loss, then to enhance the drought resistance. Furthermore, our preliminary research verified the function of *OsRLK5* to be positively related to the leaf water content and grain yield under drought stress (Figures 7, 8).

Two known genes (*OsCYP51G3* and *OsRRMh*) were mapped for grain yield in this study, and the functions of these genes correlate well with the grain yield trait. *OsCYP51G3* has been reported to mediate the biosynthesis of phytosterols and brassinosteroids, and to regulate seed setting rate (Xia et al., 2015). *OsRRMh* plays an important role during the transition from vegetable to reproductive phase in rice. Compared to the wild-type, the *OsRRMh* RNAi lines exhibit enlarged panicles while over-expression of *OsRRMh* lines lead to lower fertility rates and less number of spikelets per panicle (Liu and Cai, 2013).

Genome-wide association study (GWAS) based on large-scale re-sequencing provides a powerful tool to discover genetic variants that can be used for crop improvement, including improvement of drought resistance. GWAS has been successfully used at a high resolution to uncover associations that involve complex traits from inbred lines of field-grown maize and rice landraces and cultivars with genetic variants (Huang et al., 2010; Lu et al., 2010; Chen et al., 2014; Wen et al., 2014; Wu et al., 2015; Yano et al., 2016). Using high density SNPs at a whole-genome level, GWAS provides high-resolution genetic mapping that can narrow down the associated regions to candidate genes. Our study demonstrates that known genes as well as novel genes and loci can be dissected via GWAS analysis. Moreover, candidate genes discovered in this study provide useful information for further studies in drought resistance of crops. Functional identification of these candidate genes will improve our understanding of the mechanisms of crop responses to drought stress.

## Author contributions

LL, HL, HM, and XM designed this study. XM, KX, SC, TL, and XL performed the experiments. XM, FF, and HW analyzed the data. XM, HL, and LL drafted the manuscript.

## Funding

This work was supported by grants from the National Program for Basic Research of China (2012CB114305); the National High-Tech Research and Development Program of China (2014AA10A603); the Shanghai Municipal Commission of Science and Technology (15DZ2290700; 16ZR1431200); the Project of Subject Construction, Shanghai Academy of Agricultural Sciences, Grant No. SAAS-2016(02); the Youth Talent Development Project supported by the Shanghai Municipal Agriculture Commission (No. 2015-1-6).

### Conflict of interest statement

The authors declare that the research was conducted in the absence of any commercial or financial relationships that could be construed as a potential conflict of interest.
