# Supplementary figures and images for: Genome-Wide Association Study for Plant Height and Grain Yield in Rice under Contrasting Moisture Regimes
Source: Front Plant Sci. 2016 Nov 29;7:1801. doi: 10.3389/fpls.2016.01801 (PMC5126757; doi:10.3389/fpls.2016.01801)

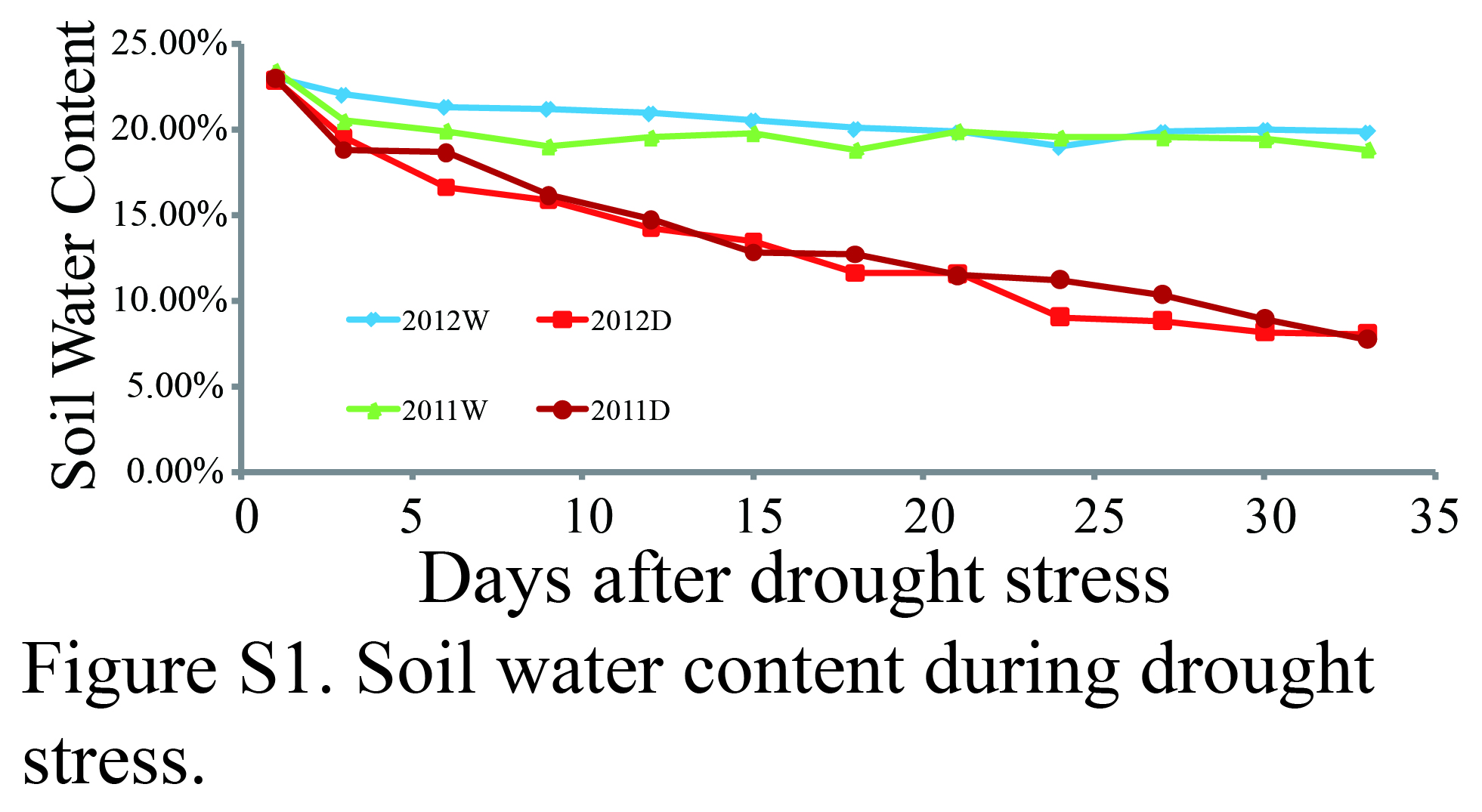

Supplement: Supplementary file 5 [file Image1.JPEG]

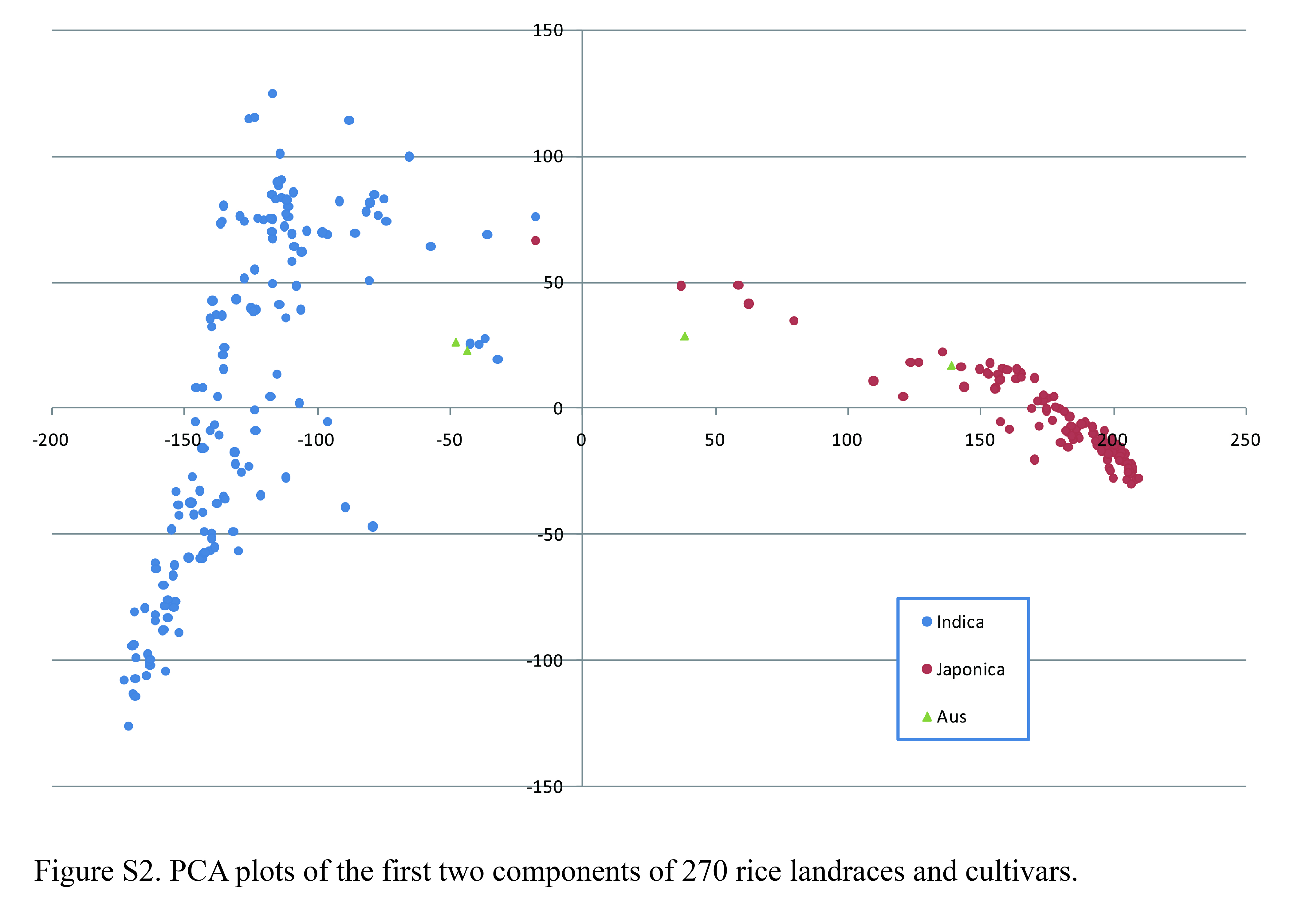

Supplement: Supplementary file 6 [file Image2.JPEG]
